# Characterization of the Efficacy of a Split Swine Influenza A Virus Nasal Vaccine Formulated with a Nanoparticle/STING Agonist Combination Adjuvant in Conventional Pigs

**DOI:** 10.3390/vaccines11111707

**Published:** 2023-11-10

**Authors:** Veerupaxagouda Patil, Juan F. Hernandez-Franco, Ganesh Yadagiri, Dina Bugybayeva, Sara Dolatyabi, Ninoshkaly Feliciano-Ruiz, Jennifer Schrock, Raksha Suresh, Juliette Hanson, Hadi Yassine, Harm HogenEsch, Gourapura J. Renukaradhya

**Affiliations:** 1 Center for Food Animal Health, Department of Animal Sciences, The Ohio State University, 1680 Madison Avenue, Wooster, OH 44691, USA; vp398@georgetown.edu (V.P.); yadaigiri.1@osu.edu (G.Y.); bugybayeva.1@buckeyemail.osu.edu (D.B.); dolatyabi.1@buckeyemail.osu.edu (S.D.); feliciano-ruiz.1@buckeyemail.osu.edu (N.F.-R.); schrock.57@osu.edu (J.S.); suresh.138@buckeyemail.osu.edu (R.S.); hanson.104@osu.edu (J.H.); 2 Department of Comparative Pathobiology, College of Veterinary Medicine, Purdue University, West Lafayette, IN 47907, USA; jfhernan@purdue.edu; 3 Biomedical Research Center, Research Institute in Doha, Qatar University, QU-NRC, Building H10, Zone 5, Room D101, Doha P.O. Box 2713, Qatar; hyassine@qu.edu.qa

**Keywords:** Nano11, ADU-S100, swine influenza A virus, intranasal vaccination, cell-mediated immune responses, memory responses, swine

## Abstract

Swine influenza A viruses (SwIAVs) are pathogens of both veterinary and medical significance. Intranasal (IN) vaccination has the potential to reduce flu infection. We investigated the efficacy of split SwIAV H1N2 antigens adsorbed with a plant origin nanoparticle adjuvant [Nano11–SwIAV] or in combination with a STING agonist ADU-S100 [NanoS100–SwIAV]. Conventional pigs were vaccinated via IN and challenged with a heterologous SwIAV H1N1-OH7 or 2009 H1N1 pandemic virus. Immunologically, in NanoS100–SwIAV vaccinates, we observed enhanced frequencies of activated monocytes in the blood of the pandemic virus challenged animals and in tracheobronchial lymph nodes (TBLN) of H1N1-OH7 challenged animals. In both groups of the virus challenged pigs, increased frequencies of IL-17A^+^ and CD49d^+^IL-17A^+^ cytotoxic lymphocytes were observed in Nano11–SwIAV vaccinates in the draining TBLN. Enhanced frequency of CD49d^+^IFNγ^+^ CTLs in the TBLN and blood of both the Nano11-based SwIAV vaccinates was observed. Animals vaccinated with both Nano11-based vaccines had upregulated cross-reactive secretory IgA in the lungs and serum IgG against heterologous and heterosubtypic viruses. However, in NanoS100–SwIAV vaccinates, a slight early reduction in the H1N1 pandemic virus and a late reduction in the SwIAV H1N1-OH7 load in the nasal passages were detected. Hence, despite vast genetic differences between the vaccine and both the challenge viruses, IN vaccination with NanoS100–SwIAV induced antigen-specific moderate levels of cross-protective immune responses.

## 1. Introduction

Swine influenza A viruses (SwIAVs) cause seasonal respiratory disease and are endemic in pig herds throughout the world, and therefore, they exert severe economic burden on the swine industry. Furthermore, both human and avian influenza viruses infect pigs, and this attribute facilitates reassortment ushering in the potential evolution of virulent virus/es exerting catastrophic effects globally. The predominant SwIAV H1N1, H1N2, and H3N2 subtypes are characterized by significant diversity in the hemagglutinin (HA), neuraminidase (NA), and the remaining six [polymerase basic protein 1 (PB1), polymerase basic protein 2 (PB2), polymerase acidic protein A (PA), nucleoprotein (NP), matrix (M), and nonstructural (NS)] genes due to antigenic drifts and shifts. Currently, the available commercial SwIAV vaccines in grow-finisher pigs display variable efficacy due to maternal antibody interference, an inability to protect against variants, and inadequate mucosal adaptive immune responses. Hence, SwIAVs pose a challenge, and therefore, novel effective mucosal vaccines are needed for effective control and mitigation strategies [1,2].

Antigen-specific cell-mediated immune responses against influenza A viruses (IAV) are of critical significance. Unlike neutralizing antibodies, T cells targeting conserved epitopes and nucleoprotein (NP) epitopes play a significant role in protective cell-mediated immunity [3,4]. The predominant H3N2 NP-specific T cell responses during seasonal and pandemic flu outbreaks during 2006 to 2010 were associated with robust cross-protection in the absence of protective antibody responses [3]. A cross-reactive cluster of differentiation 8 positive T-lymphocyte (CD8^+^ T cell) response has a significant protective role in heterologous clinical and preclinical prime/challenge studies between H1N1, H7N7, H5N1, and H3N2 influenza viruses [5,6,7,8]. Furthermore, a universal influenza vaccine candidate in pigs mitigated the lung pathology and reduced the nasal and lung viral load of homologous and heterologous challenge viruses in the absence of neutralizing antibodies [9]. Intranasal administration of pandemic H1N1 IAV peptides and inactivated H1N2 IAV-encapsulated poly D, L-lactic-*co*-glycolic acid (PLGA) nanoparticle vaccine elicited a robust antigen-specific T cell immunity despite the lack of neutralizing humoral immunity in pigs [10]. Hence, the present project studied the safeguarding role/s of antigen-specific cell-mediated memory repertoire in conventional pigs.

Nanoparticle-based vaccines offer many advantages leading to efficacious antigen presentation and robust immunogenicity [11,12]. Earlier publications from our group demonstrated the significance of corn-based cationic alpha-D-glucan nanoparticles (Nano11) in mice and pigs, showing increased immunogenicity to vaccine antigens in mice and reduced virus load in pigs [13]. The intrinsic adjuvanticity of Nano11 can be augmented via the adsorption of suitable adjuvants such as Toll-like receptor-3 (TLR3) agonist poly(I:C) and the STING-agonist cyclic-di-adenosine monophosphate (AMP) [13]. Inactivated split IAV vaccines are more efficacious than inactivated whole virus vaccines [14,15,16,17]. Earlier studies have demonstrated that the split influenza virus vaccine rapidly and potently activates multiple immune cell types, and splitting increases vaccine safety [18]. Currently, the split virus format is the most prevalent influenza vaccine (Fluzone; Sanofi Pasteur) prescribed for adults and is distributed in the United States administered without adjuvant [19]. Therefore, with the goal of further improving the efficacy of our Nano11 vaccine delivery platform, the current study assessed the efficacy of an intranasally administered combination of split virus SwIAV H1N2 antigens comprising ADU-S100, a synthetic equivalent counterpart of cyclic-di-AMP, co-adsorbed onto Nano11 (NanoS100–SwIAV) in conventional swine. The vaccine efficacy was evaluated by challenge with virulent heterologous SwIAV H1N1-OH7 and H1N1 2009 pandemic viruses.

## 2. Materials and Methods

### 2.1. Vaccines and Challenge Viruses

SwIAV H1N2-OH10 (A/Swine/OH/FAH10-1/10) [20], a δ lineage virus, was used to prepare the vaccine antigen by Binary ethylenimine (BEI) inactivation and detergent (1% Triton-X100 + 0.05% Tween-80) splitting whereas SwIAV H1N1-OH7 (A/Swine/OH/24366/2007), a γ lineage triple reassortant virus [21], and H1N1 2009 pandemic (A/California/04/2009) [22] were used as challenge viruses. In addition, a heterosubtypic IAV H3N2-OH4 (A/Turkey/OH/313053/2004) [23] was employed for the assessment of cross-reactive humoral response. The vaccine SwIAV H1N2-OH10 virus is heterologous to both the challenge viruses, with over 77% HA gene homology. In addition, the SwIAV H1N1-OH7 and H1N1 pandemic challenge viruses are heterologous to each other (93.5% HA gene identity). The SwIAV were grown in Madin–Darby canine kidney (MDCK) cells. A mutant variety of sweet corn was used to prepare Nano11 as previously described [13]. The killed SwIAV split antigen was adsorbed onto Nano11 with or without the synthetic STING agonist ADU-S100 (ChemieTek, Indianapolis, IN, USA) to generate NanoS100–SwIAV or Nano11–SwIAV vaccines, respectively.

### 2.2. Characterization of NanoS100–SwIAV or Nano11–SwIAV

Ultraperformance liquid chromatography/tandem mass spectrometry was employed to characterize the adsorption efficiency of ADU-S100 to Nano11 [24]. Nano11 was incorporated with ADU-S100 for 1 h at 4 °C, and the mixture was subjected to centrifugation at 20,800× *g* for 15 min at 4 °C. The upper liquid phase was harvested and shifted to a tube with a 300 kDa membrane (Pall, New York, NY, USA). The sample was centrifuged at 600× *g* for 30 min at 4 °C. The flow-through sample was processed by using Agilent 1200 HPLC system connected to an Agilent 6460 triple-quadrupole mass spectrometer (Agilent Technologies, San Jose, CA, USA) to determine the concentration of the adsorbed ADU-S100 (MIW815) onto Nano11. Nano11 and killed SwIAV split antigen were electrostatically adsorbed. A Zetasizer (Nano ZS90, Malvern, UK) was employed to determine polydispersity leading to the measurement of the particle size, charge, and molecular weight of Nano11 ± ADU-S100 ± SwIAV split antigen by employing dynamic light scattering.

### 2.3. Animal Studies

**Figure 1:**
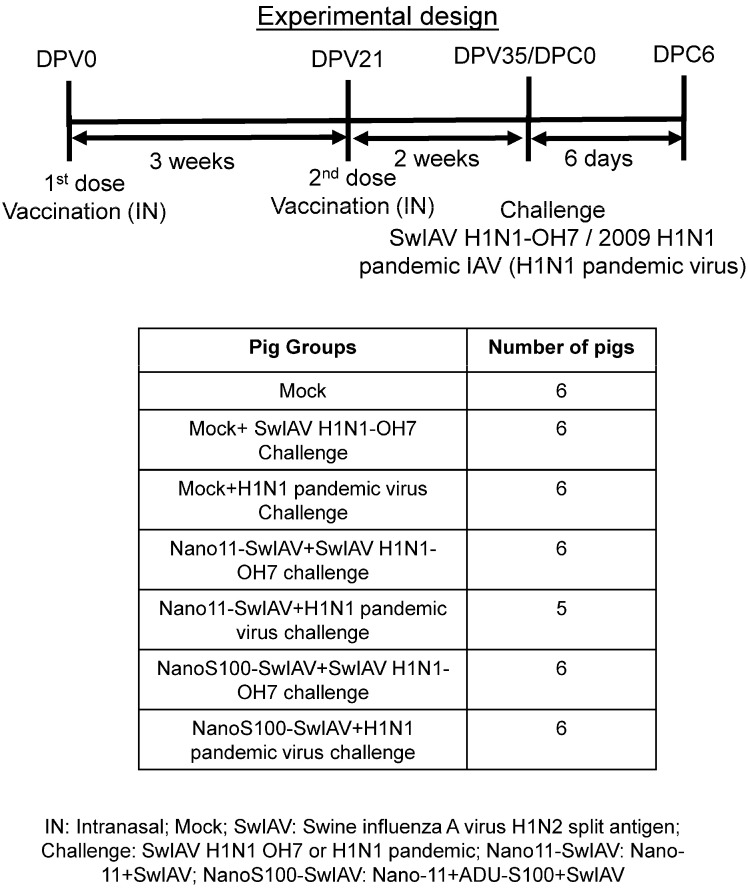
Experimental plan to ascertain the NanoS100–SwIAV vaccine efficacy in pigs. Conventional pigs were immunized twice with Nano11–SwIAV or NanoS100–SwIAV split virus vaccine or controls Mock IN and challenged at day 35 post-prime vaccination with SwIAV H1N1-OH7 or H1N1 pandemic virus and euthanized at day 6 post-challenge (DPC6). Nasal swabs, blood, lungs, tracheobronchial lymph nodes [TBLN], and bronchioalveolar [BAL] lavage fluid samples were harvested for the analysis. Five–six pigs were used in each of the experimental groups.

Four-week-old conventional piglets were procured and housed in the isolation facility. The animals were randomly assigned to the indicated experimental groups. These pigs were vaccinated twice intranasally using a custom made multidose device (IN; spray mist delivery device [Prima Tech, Kenansville, NC, USA] (refer to device picture [25]. A vaccine volume of 0.5 mL in DMEM per nostril (a total of 1 mL per pig) containing NanoS100–SwIAV, Nano11–SwIAV, or control saline (Mock) were administered and then challenged with SwIAV H1N1-OH7 or H1N1 pandemic virus [2 × 10^7^ tissue culture infective dose 50 (TCID_50_/mL) per pig at post-prime vaccination day 35; half the virus amount was delivered intratracheal and the remaining half the dose IN] (Figure 1). The vaccine antigen was quantified and validated by standard procedures such as bicinchoninic acid (BCA) assay for protein estimation, and the hemagglutination (HA) units were determined. Each 1 mL vaccine included 0.5 mg Nano11, 50 µg ADU-S100, and 250 µg split viral protein consisting of 50 HA units. Animals were euthanized at day 6 post-challenge (DPC6) to determine the antigen-specific memory immune responses. Blood and nasal swab samples were harvested on day 0 post-vaccination (DPV0), DPV21, and day 0 of challenge (DPC0). On the day of necropsy on DPC6, blood, nasal swab, lung, TBLN, and bronchoalveolar lavage (BAL) fluid samples were collected. Furthermore, nasal swab samples were obtained on DPC0, DPC2, DPC4, and DPC6.

### 2.4. Surface and Intracellular Cytokine Labeling for Flow Cytometry

Peripheral blood mononuclear cells (PBMCs), TBLN cells, and BAL cells were harvested, restimulated, and immunostained as described previously [17]. Five million immune cells were cultured in each well of a 48-well flat bottom plate in 1 mL of medium (RPMI 1640 10% FBS) containing SwIAV H1N1-OH7 or H1N1 pandemic virus at 0.1 multiplicity of infection (MOI) and recombinant porcine IL-2 for 48 h (hr) in vitro. For the last 6 h of the incubation, cells in culture were treated with protein transport inhibitor Brefeldin A (GolgiPlug). Cells were collected and washed. Blocking was performed with 1% normal rabbit serum. An equal number of cells were dispensed into the required number of wells in a 96-well round bottom plate for the downstream flow cytometry experiments. Indicated isotype control antibodies were incorporated as negative controls. The flow cytometry antibody panels and their corresponding isotype antibodies are explained in Supplementary Tables S1–S3. When cells were labeled with a purified monoclonal antibody (mAb), its corresponding secondary antibody was added and then blocked with 1% normal mouse serum. Subsequently, the cells were labeled with other cell surface markers added as a cocktail. The cells were washed twice in 200 µL FACS buffer/well. The surface labeling was carried out by using fluorochrome conjugated mAbs or their corresponding isotype controls at previously determined concentrations in 50 µL FACS buffer for 30 min at 4 °C. Cells were subjected to fixation using 1% PFA at 4 °C for 30 min and resuspended in 200 µL FACS buffer.

Intracellular labeling procedure: Cells washing was performed once and permeabilization was performed with 1% saponin for 45 min at room temperature. This was followed by washing with saponin wash buffer (0.1% saponin). Incubation with fluorochrome conjugated mAbs or their corresponding isotype antibodies in 50 µL final volume of saponin wash containing 1% normal rabbit serum for 45 min at 4 °C was carried out subsequently. Cells were washed once in the saponin wash. Labeling with indicated secondary antibodies for 45 min at 4 °C was carried out in the next step. Cell washing was performed once, and the cells were resuspended in 200 µL FACS buffer and transferred to FACS tubes. Acquisition of the samples was performed using a live cell gate in a BD FACS Aria II flow cytometer. For each sample, 100,000 events were collected. The flow cytometry data were investigated using FlowJo software (FlowJo V10, Becton, Dickinson & Company, BD; Franklin Lakes, NJ, USA), and data plotting was carried out by employing GraphPad Prism (GraphPad Prism 9, San Diego, CA, USA). In all of the flow cytometry labeling panels, isotype and fluorescence minus one (FMO) control was maintained for representative pigs in each of the experimental groups. These controls were used for determining the gating strategy to differentiate positive and negative populations for the indicated markers.

### 2.5. Enzyme-Linked Immunosorbent Assay (ELISA)

SwIAV-specific immunoglobulins IgG and secretory IgA (sIgA) antibodies titers were characterized [25]. High binding affinity 96-well flat bottom plates were employed for ELISA. The concentrations of the killed virus antigens were optimized by titration experiments earlier. Those concentrations of SwIAV H1N1-OH7, H1N2-OH10, or H3N2-OH4 were employed for coating. The coated plates were incubated overnight at 4 °C. The plates were washed three times with PBS-Tween20 (0.05%) (PBST). Blocking with 5% dry milk in PBST was carried out at room temperature (RT) for 2 h. Test samples were serially diluted in 2.5% dry milk powder in PBST at indicated dilutions [1:2 for nasal swab, 1:500 for serum, and 1:50 for BAL and lung lysate specimens]. The diluted samples (50 μL/well) were added to plates and incubated overnight at 4 °C. After the washing step, the plates were incubated with 50 μL/well of goat anti-pig IgA conjugated with HRP (Bethyl Laboratories Inc., Montgomery, TX, USA) its secondary antibody at the previously standardized 1:2000 dilution or peroxidase labeled AffiniPure goat anti-swine IgG (H+L) (Jackson ImmunoResearch Laboratories Inc., West Grove, PA, USA) at 1:8000 dilution in 2.5% dry milk in PBST. The plates were washed, and incubation was carried out with a 1:1 mixture of peroxidase substrate solution B and TMB (KPL, MD) (50 μL/well) for 10–20 min at RT. Phosphoric acid (1 molar [M]) (50 μL/well) was used as a stop solution. The optical density (OD) was documented by a Spectramax microplate reader at 450 nm. The corrected OD values were calculated by subtracting the average value of blank from the test samples.

### 2.6. Virus Titration

Virus titers were characterized as per optimized lab protocol [25]. MDCK (2 × 10^4^ cells/well) were cultured in 200 μL DMEM complete media. Incubation was carried out in a 37 °C humidified 5% CO_2_ incubator overnight. Round bottom 96-well plates were used for preparing ten-fold serial dilutions of nasal swab, BAL fluid, and lung lysate samples in serum-free DMEM. The plates with confluent monolayers of MDCK cells were washed with PBS. Cells were inoculated with 100 μL/well of serially diluted test samples for 1.5 h at 37 °C in a 5% CO_2_ incubator. One hundred microliters of DMEM serum-free medium with 2 µg/mL L-(tosylamido-2-phenyl) ethyl chloromethyl ketone (TPCK)-trypsin (Sigma, St. Louis, MO, USA) was added to the wells. Plates were incubated at 37 °C in humidified 5% CO_2_ incubator for 36 h. The cells were fixed using 80% acetone (10 min). Labeled with an influenza A virus (IAV) NP protein specific mAb (#M058, CalBioreagents, San Mateo, CA, USA), and diluted at 1:5000 for 2 h at 37 °C in a 5% CO_2_ incubator. Plates were washed and incubated for 1.5 h with 1:3000 dilution of (50 μL/well) the secondary antibody Alexa Fluor 488 conjugated with goat anti-mouse IgG (H+L) antibody (Life Technologies, Carlsbad, CA, USA) at 37 °C in a 5% CO_2_ incubator. Glycerol was mixed with PBS, pH = 8 at 6:4 proportion, and used as the mounting medium. Infection was documented using an Olympus fluorescent microscope (Olympus, Center Valley, PA, USA). Infectious titer was calculated by the Reed and Muench method. For live virus detection, by using immunofluorescence assay, the cutoff was 1:5 dilution of the samples, which gives background fluorescence, and anything 1 log_10_ and above was considered specific.

### 2.7. Statistical Analysis

Statistical analysis of flow cytometry data was assessed by using one-way ANOVA [one independent variable: conventionally raised pigs divided into groups representing different independent treatments] followed by Tukey’s post-test. Analysis of the titers of IgG and sIgA antibody responses was performed using two-way ANOVA [two independent variables: conventionally raised pigs divided into groups representing different independent treatments and different independent dilutions of samples] followed by the Bonferroni post-test. Data indicate the mean value of 6 pigs ± SEM (standard error of the mean).

## 3. Results

### 3.1. Characterization of the Vaccine Formulations

The Nano11 surface adsorbed split SwIAV H1N2 Ags and ADU-S100 adjuvant; either together (NanoS100–SwIAV) or only the virus antigen (Nano11–SwIAV) were characterized for their polydispersity index (PDI), adsorption efficiency, size, and zeta potential in our recently published article [17].

### 3.2. NanoS100–SwIAV Vaccine Partially Decreased the Challenge Virus Titers in the Respiratory Tract of Conventional Pigs

**Figure 2:**
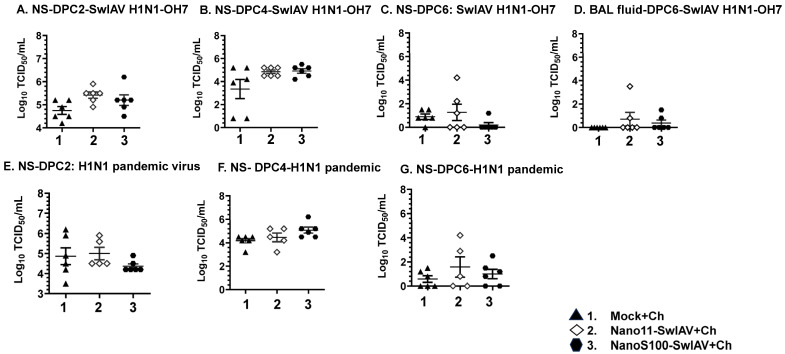
Infectious challenge virus titers in the nasal passage of intranasally (IN) vaccinated pigs. Conventional pigs were immunized twice with Nano11–SwIAV or NanoS100–SwIAV split virus vaccine or controls Mock IN, challenged at day 35 post-prime vaccination with SwIAV H1N1-OH7 or H1N1 pandemic virus, and euthanized at day 6 post-challenge (DPC6). The titers of challenge viruses were characterized by using cell culture method in nasal swab (NS) at (**A**,**E**) DPC2; (**B**,**F**) DPC4; (**C**,**G**) DPC6, and (**D**) BAL fluid at DPC6. Data indicate the mean value of 5 or 6 pigs ± SEM. Statistical analysis was carried out by one-way ANOVA followed by Tukey’s post-test.

We monitored the clinical signs of flu (rectal temperature, lethargy, and anorexia) post-challenge and scored the gross lung lesions of pigs during necropsy. However, neither of the challenge viruses caused appreciable clinical signs of disease, and during necropsy did not observe any difference among the pig groups with respect to lung lesions scores. This is likely because pigs were euthanized at DPC6. To appreciate any differences in the lung lesions, DPC2 to DPC4 is ideal during the active infection. Further, the safeguarding potential of the Nano11–SwIAV and NanoS100–SwIAV IN vaccines was assessed by measuring the titers of the challenge SwIAV H1N1-OH7 and H1N1 pandemic virus in the nasal swabs (NS), BAL fluid, and lung lysate specimens. A remarkable abatement of SwIAV H1N1-OH7 titers (not significant) in the nasal passages of the NanoS100–SwIAV vaccinates compared to the Mock+Ch group animals was observed at DPC6 (Figure 2C) but not at DPC2 and 4 (Figure 2A,B). In addition, the challenge virus titers were very low in BAL fluid (Figure 2D). In the H1N1 pandemic virus-challenged NanoS100–SwIAV vaccinates, we observed a reduced trend (not significant) in the nasal viral load at DPC2 (Figure 2E), but not at DPC4 and 6 (Figure 2F,G). Taken together, these observations corroborated that the intranasally delivered NanoS100–SwIAV vaccine conferred partial cross-protection in the upper respiratory tract of conventional pigs.

### 3.3. Both Nano11–SwIAV and NanoS100–SwIAV Vaccines Downregulated the Frequencies of Activated Dendritic Cells (CD3^−^CD172a^+^SynCAM^+^CD80/86^+^) and Enhanced the Activated Monocytes (CD3^−^CD172a^+^SynCAM^−^CXCL10^+^CD80/86^+^) in Both Mucosal and Systemic Compartments of Vaccinates at DPC6

In order to understand the mechanisms of the immune responses, innate immune cell frequencies in TBLN mononuclear cells (MNCs) and PBMCs were analyzed. The gating strategy employed for the identification of dendritic cells and monocytes is described in Supplementary Figure S1A. Larger and more granular cells were gated, and after the exclusion of doublets and CD3^+^ lymphocytes, CD172a^+^SynCAM^+^ cells were considered as putative porcine dendritic cells and CD172a^+^SynCAM^−^ cells as monocytes [26]. Different types of cells, including myeloid cells, express an IFNγ-inducible protein 10 called CXCL10, which is also called T-helper type-1 (Th-1) chemokine. Hence, CXCL10 expression can be used as a marker for the induction of innate immunity (34; 35). The activated antigen-presenting cells (APCs) upregulate the cell surface expression of CD80 (B7-1) and CD86 (B7-2). In addition, they deliver a second signal to T cells when they engage their ligands CD28 and CD152 (CTLA-4), and expression of CD80/86 on pig dendritic cells and monocytes specifies their activation status [27]. Therefore, we investigated the frequencies of CXCL10^+^ and CD80/86^+^ dendritic cells and monocytes in both lung draining TBLN and PBMCs of pigs vaccinated intranasally and challenged with influenza viruses.

**Figure 3:**
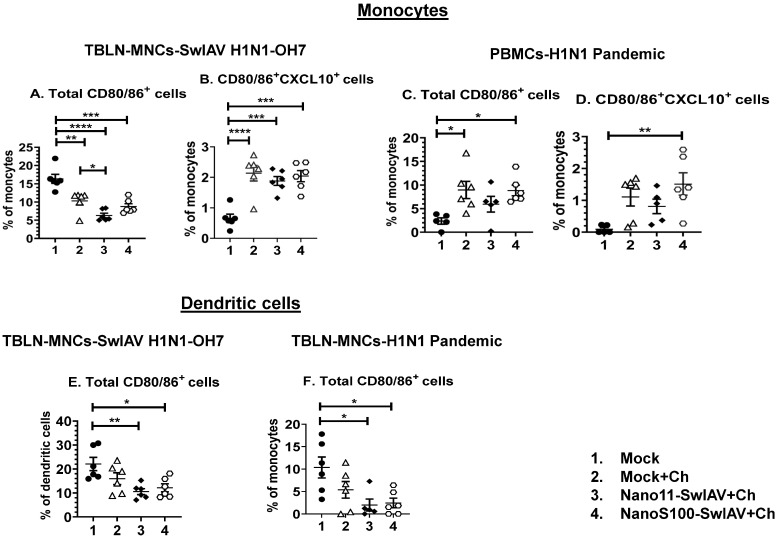
The candidate Nano11-based vaccines modulated the activation of innate myeloid immune cell frequencies in vaccinated pigs. Conventional pigs were immunized twice with Nano11–SwIAV or NanoS100–SwIAV split virus vaccine or controls Mock IN, challenged at day 35 post-prime vaccination with SwIAV H1N1-OH7 or H1N1 pandemic virus, and euthanized at day 6 post-challenge (DPC6). TBLN MNCs and PBMCs were purified at DPC6 and were incubated with SwIAV H1N1-OH7 or H1N1 pandemic in vitro to determine memory responses. The cells were immunolabeled and flow cytometry was employed to investigate the frequencies of dendritic cells (CD3^−^CD172a^+^SynCAM^+^) and monocytes (CD3^−^CD172a^+^SynCAM^−^). In SwIAV H1N1-OH7-challenged pigs, TBLN-MNCs were analyzed: (**A**) total CD80/86^+^ monocytes; (**B**) CD80/86^+^CXCL10^+^ monocytes and H1N1 pandemic virus-challenged pigs PBMCs; (**C**) total CD80/86^+^ monocytes; and (**D**) CD80/86^+^CXCL10^+^ monocytes. Dendritic cells in (**E**) SwIAV H1N1-OH7-challenged TBLN-MNCs total CD80/86^+^ and (**F**) H1N1 pandemic virus-challenged TBLN MNCs total CD80/86^+^ cells were characterized. Data represent the mean value of 5–6 pigs ± SEM. Statistical analysis was carried out by one-way ANOVA followed by Tukey’s post-test. Asterisks indicate significant difference between indicated groups (* *p* < 0.05, ** *p* < 0.01, *** *p* < 0.001, **** *p* < 0.0001).

Monocytes (CD3^−^CD172a^+^SynCAM^−^): In TBLN MNCs challenged with SwIAV H1N1-OH7, the frequencies of CD80/86^+^ monocytes were decreased significantly, while activated monocytes expressing both CXCL10 and CD80/86 were significantly increased compared to the mock group in all of the animal groups (*p* < 0.01 to *p* < 0.001) (Figure 3A,B). However, a significant enhancement of both CXCL10 and CD80/86 in monocytes of the NanoS100–SwIAV+Ch group was detected compared to the mock animals in PBMCs of H1N1 pandemic virus-challenged pigs (*p* < 0.05 to *p* < 0.01) (Figure 3C,D). In addition, the Nano11–SwIAV+Ch animals exhibited significantly decreased frequencies of total CD80/86^+^ monocytes compared to the Mock+Ch group (*p* < 0.05) in TBLN MNCs challenged with SwIAV-OH7 (Figure 3A).

Dendritic cells (CD3^−^CD172^+^SynCAM^+^): the frequencies of total CD80/86^+^ dendritic cells were significantly decreased in both the Nano11–SwIAV+Ch and NanoS100–SwIAV+Ch group of animals compared to the mock group (*p* < 0.05 to *p* < 0.01) in TBLN MNCs upon challenge with both SwIAV H1N1-OH7 (Figure 3E) and H1N1 pandemic virus (Figure 3F).

Hence, the above results indicate that both Nano11–SwIAV and NanoS100–SwIAV vaccines induced moderate activation of innate immune responses in the draining TBLN and systemically in vaccine-administered conventional pigs at DPC6.

### 3.4. Both the Candidate Nano11-Based Vaccines Elicited Strong Virus-Specific IL-17A and IFNγ Secreting Recall CTL Responses in The Lung Draining TBLN of Vaccinates

**Figure 4:**
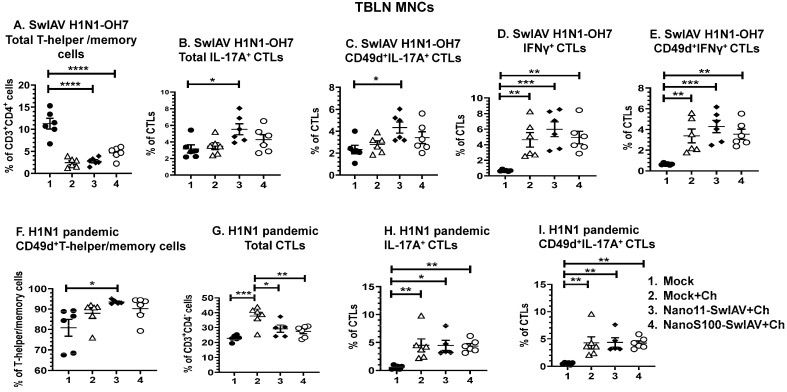
The candidate Nano11-based vaccines elicited IL-17A^+^ and IFNγ^+^ CTL cell responses in TBLN MNCs of vaccinated pigs. Conventional pigs were immunized twice with Nano11–SwIAV or NanoS100–SwIAV split virus vaccine or controls Mock IN, challenged at day 35 post-prime vaccination with SwIAV H1N1-OH7 or H1N1 pandemic virus, and euthanized at day 6 post-challenge (DPC6). TBLN MNCs isolated at DPC6 were restimulated with SwIAV H1N1-OH7 or H1N1 pandemic virus in vitro. The SwIAV H1N1-OH7-restimulated TBLN MNCs were immunolabeled and analyzed by flow cytometry for the frequencies of the following: (**A**) total T-helper/memory cells [CD3^+^CD4^+^CD8α^+^CD8β^−^]; (**B**) total IL-17A^+^CTLs [CD3^+^CD4^−^-CD8α^+^CD8β^+^ IL-17A^+^]; (**C**) CD49d^+^IL-17A^+^ CTLs [CD3^+^CD4^−^CD8α^+^CD8β^+^IL-17A^+^CD49d^+^]; (**D**) total IFNγ^+^ CTLs [CD3^+^CD4^−^CD8α^+^CD8β^+^ IFNγ^+^]; and (**E**) CD49d^+^IFNγ^+^ CTLs. The H1N1 pandemic virus-restimulated TBLN MNCs were immunolabeled and analyzed by flow cytometry for the frequencies of the following: (**F**) CD49d^+^T-helper/memory cells [CD3^+^CD4^+^CD8α^+^CD8β^−^]; (**G**) total CTLs [CD3^+^CD4^−^CD8α^+^CD8β^+^]; (**H**) total IL-17A^+^ CTLs [CD3^+^CD4^−^CD8α^+^CD8β^+^IL-17A^+^]; and (**I**) CD49d^+^IL-17A^+^ CTLs [CD3^+^CD4^−^CD8α^+^CD8β^+^CD49d^+^IL-17A^+^]. Data represent the mean value of 5 or 6 pigs ± SEM. Statistical analysis was performed by one-way ANOVA followed by Tukey’s post-test. Asterisks represent significant difference between indicated groups (*p < 0.05, ** *p* < 0.01, *** *p* < 0.001, **** *p* < 0.0001).

The memory T cells responses in TBLN were characterized by multi-color flow cytometry (Figure 4A–I), and the standard gating strategy was followed to detect CD49d^+^IL-17A^+^ and IFNγ^+^ T-helper/memory cells and cytotoxic T lymphocytes (CTLs) (Supplementary Figure S1B,C). The expression of the CD49d integrin was increased upon activation of T cells and can be used as a surrogate marker for antigen-experienced or memory T cells [28]. The frequencies of total T-helper/memory cells were significantly downregulated (*p* < 0.0001) in all of the SwIAV H1N1-OH7-challenged pigs compared to the mock animals (Figure 4A). The TBLN MNCs of Nano11–SwIAV vaccinates upon challenge with SwIAV H1N1-OH7 had significantly augmented frequencies of IL-17A^+^ CTLs and CD49d^+^IL-17A^+^ CTLs compared to the mock group (*p* < 0.05) (Figure 4B,C), While both Nano11–SwIAV and NanoS100–SwIAV vaccinates and the Mock+Ch group animals challenged with SwIAV H1N1-OH7 exhibited a significant increase in the percentage of IFNγ^+^ CTLs and CD49d^+^IFNγ^+^ CTLs compared to the mock group (*p* < 0.01 to *p* < 0.001) (Figure 4D,E).

In the H1N1 pandemic virus challenge, Nano11–SwIAV vaccinates displayed a significant enhancement of the frequencies of CD49d^+^ Th/memory cells compared to the mock group animals (*p* < 0.05) (Figure 4F); however, the frequencies of total CTLs were significantly upregulated (*p* < 0.05 to *p* < 0.0001) in all of the H1N1 pandemic virus-challenged pigs compared to the mock animals (Figure 4G). A significant increase in the frequencies of IL-17A^+^ CTLs (*p* < 0.05 to *p* < 0.01) (Figure 4H) and CD49d^+^IL-17A^+^ CTLs (*p* < 0.01) (Figure 4I) compared to the mock group animals was observed in all of the H1N1 pandemic virus-challenged animals. These observations underline the ability of both of the Nano11-based vaccine formulations to elicit moderate induction of antigen-specific IL-17A secreting memory T cells in the draining TBLN of vaccinates at DPC6.

### 3.5. Both of the Candidate Nano11 Vaccines Elicited the Challenge Virus-Specific IFNγ Secreting T Cell Responses in PBMCs of Vaccinated Pigs

**Figure 5:**
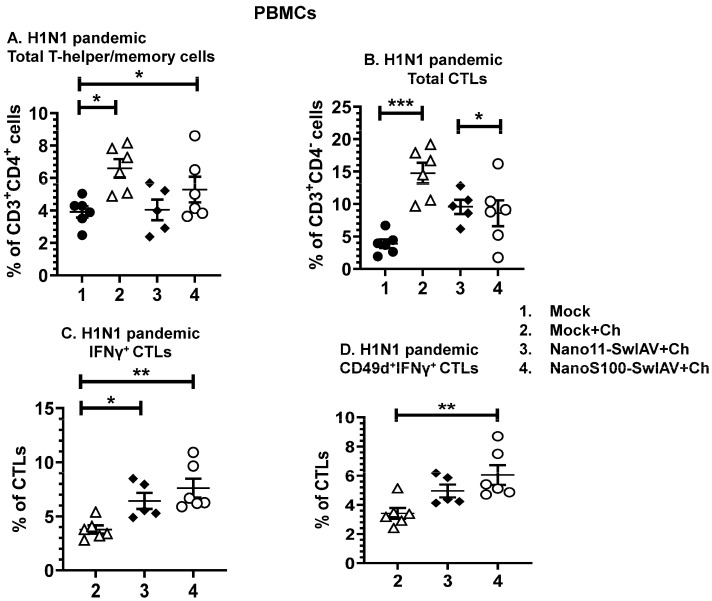
NanoS100–SwIAV and Nano11–SwIAV vaccines augmented IFNγ^+^ CTL frequencies in PBMCs of vaccinated pigs. Conventional pigs were immunized twice with Nano11–SwIAV or NanoS100–SwIAV split virus vaccine or controls Mock IN, challenged at day 35 post-prime vaccination with SwIAV H1N1-OH7 or H1N1 pandemic virus, and euthanized at day 6 post-challenge (DPC6). H1N1 pandemic challenge virus was used for the restimulation of DPC6 isolated PBMCs in vitro. The cells were analyzed for the determination of the frequencies of the following: (**A**) total T-helper/memory cells [CD3^+^CD4^+^CD8α^+^CD8β^−^]; (**B**) total CTLs [CD3^+^CD4^−^CD8α^+^CD8β^+^]; (**C**) IFNγ^+^ CTLs [CD3^+^CD4^−^CD8α^+^CD8β^+^IFNγ^+^]; and (**D**) CD49d^+^IFNγ^+^ CTLs [CD3^+^CD4^−^CD8α^+^CD8β^+^IFNγ^+^CD49d^+^). Data display the mean value of 5–6 pigs ± SEM. Statistical analysis was conducted by using one-way ANOVA followed by Tukey’s post-test. Asterisks show significant difference between indicated groups (* *p* < 0.05, ** *p* < 0.01, *** *p* < 0.001).

Vaccination with the NanoS100–SwIAV vaccine as well as in the mock animals had significantly upregulated frequencies of T-helper/memory cells in PBMCs following H1N1 pandemic virus challenge infection compared to the mock control group (*p* < 0.05) (Figure 5A). While the frequencies of CTLs were significantly increased in the Mock+Ch group animals compared to the mock group (*p* < 0.001) (Figure 5B). However, the Nano11–SwIAV vaccinated animals exhibited downregulated frequencies of total CTLs compared to Nano11–SwIAV vaccinates in H1N1 pandemic virus-challenged animals (*p* < 0.05) (Figure 5B).

In contrast, intranasal administration of Nano11–SwIAV and NanoS100–SwIAV vaccines elicited a significant augmentation of the frequencies of H1N1 pandemic virus-specific IFNγ^+^CTLs compared to the Mock+Ch group animals (*p* < 0.05 to *p* < 0.01) (Figure 5C). In addition, CD49d^+^IFNγ^+^ CTLs were enhanced significantly **(***p* < 0.01) in NanoS100–SwIAV vaccinates (Figure 5D) systemically. These observations suggest that the NanoS100–SwIAV vaccine elicited systemic challenge virus-specific CTL recall responses in vaccinated conventional pigs at DPC6.

### 3.6. Both of the Candidate Vaccines in SwIAV-H1N1-OH7-Challenged Pigs Elicited Cross-Reactive Virus-Specific Mucosal Antibody Response in Vaccinates at DPC6

**Figure 6:**
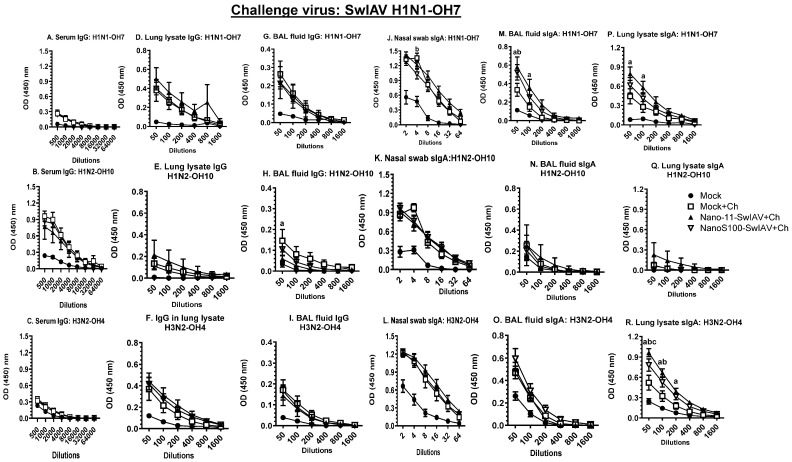
NanoS100–SwIAV and Nano11–SwIAV vaccines upregulated the SwIAV H1N1-OH7-specific sIgA responses in the lungs of vaccinated pigs. Conventional pigs were immunized twice with Nano11–SwIAV or NanoS100–SwIAV split virus vaccine or controls Mock IN and challenged at day 35 post-prime vaccination with SwIAV H1N1-OH7. The vaccinates were subjected to necropsy at day 6 post-challenge (DPC6). Serum, lung lysate, and BAL fluid specimens collected at DPC6 were investigated by ELISA to detect antigen-specific IgG antibody responses against SwIAV (**A**,**D**,**G**) H1N1-OH7; (**B**,**E**,**H**) H1N2-OH10; (**C**,**F**,**I**) H3N2-OH4 and to detect antigen-specific sIgA antibody responses in nasal swab, BAL fluid, and lung lysate samples against SwIAV (**J**,**M**,**P**) H1N1-OH7; (**K**,**N**,**Q**) H1N2-OH10; (**L**,**O**,**R**) H3N2-OH4. Data exhibit the mean value of 5–6 pigs ± SEM. Statistical analysis was conducted by using two-way ANOVA followed by Bonferroni post-test. Letters a, b, and c indicate significance between Mock+Ch versus Nano11–SwIAV+Ch, Mock+Ch versus NanoS100–SwIAV+Ch, and Nano11–SwIAV+Ch versus NanoS100–SwIAV+Ch, respectively, at the indicated sample dilutions.

Animals vaccinated with NanoS100–SwIAV and Nano11–SwIAV vaccines and challenged with the heterologous SwIAV H1N1-OH7 exhibited significantly augmented SwIAV H1N1-OH7- and H3N2-OH4-specific sIgA antibody titers in lung lysate and BAL fluid compared to the Mock+Ch animals (Figure 6M,P,R), respectively, at DPC6. These results corroborated that upon SwIAV-H1N1-OH7 challenge, both of the Nano11 vaccines induced SwIAV-specific heterologous and heterosubtypic sIgA titers in lungs. However, non-vaccinated and SwIAV H1N1-OH7-challenged pigs had comparable titers of IgG and IgA antibody responses in the serum and nasal swab to those of both of the Nano11 vaccinates (Figure 6A–C,H,J,K). This was also evident at DPC0 in the mock animals (Supplementary Figure S2), confirming the presence of high levels of pre-existing maternal antibodies in conventional pigs used in the study.

### 3.7. NanoS100–SwIAV Vaccine Elicited Both Cross-Reactive H1N1 Pandemic Virus-Specific Lung and Systemic IgG and Lung sIgA Responses in Vaccinates at DPC6

**Figure 7:**
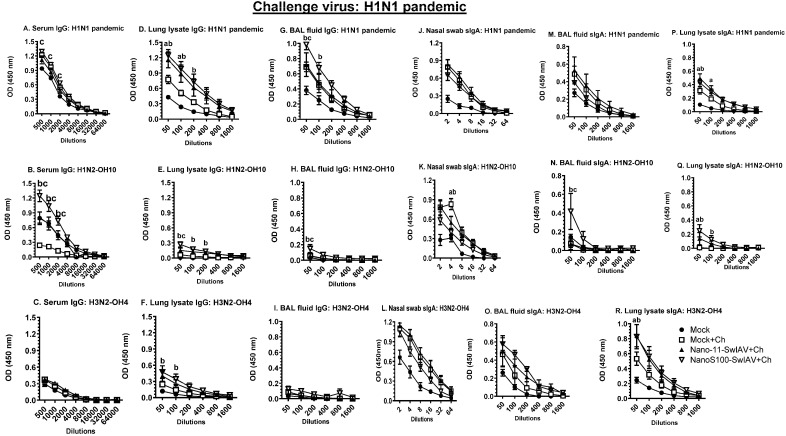
NanoS100–SwIAV increased the H1N1 pandemic-specific IgG and sIgA responses in both lungs and blood of vaccinated pigs. Conventional pigs were immunized twice with Nano11–SwIAV or NanoS100–SwIAV split virus vaccines or control Mock IN and challenged at day 35 post-prime vaccination with H1N1 pandemic virus. The experimental animals were sacrificed at day 6 post-challenge (DPC6). Serum, lung lysate, and BAL fluid samples harvested at DPC6 were investigated by ELISA to detect IgG antibody responses against SwIAV (**A**,**D**,**G**) H1N1 pandemic; (**B**,**E**,**H**) H1N2-OH10; (**C**,**F**,**I**) H3N2-OH4 and to determine sIgA antibody responses in nasal swab, BAL fluid, and lung lysate samples against SwIAV (**J**,**M**,**P**) H1N1 pandemic; (**K**,**N**,**Q**) H1N2-OH10; (**L**,**O**,**R**) H3N2-OH4. Data show the mean value of 5–6 pigs ± SEM. Statistical analysis was conducted by using two-way ANOVA followed by Bonferroni post-test. Letters a, b, and c display the significance between Mock+Ch versus Nano11–SwIAV+Ch, Mock+Ch versus NanoS100–SwIAV+Ch, and Nano11–SwIAV+Ch versus NanoS100–SwIAV+Ch, respectively, at the indicated sample dilutions.

The antigen-specific IgG and sIgA titers in serum, lung lysate, and BAL fluid samples of vaccinates were determined at DPC6 (Figure 7A–R). NanoS100–SwIAV vaccine induced significantly higher serum, BAL fluid, and lung lysate IgG titers against H1N1pandemic, SwIAV H1N2-OH10, and H3N2-OH4 compared to the mock vaccinated virus-challenged animals (Figure 7A,B,D–G). Similarly, in NanoS100–SwIAV vaccinates, a significant enhancement of H1N1 pandemic and SwIAV H1N2-OH10- and H3N2-OH4-specific sIgA titers in lung lysate and BAL fluid was observed compared to the mock vaccinated virus-challenged animals (Figure 7P–R), respectively, at DPC6. These data corroborated that a combination of Nano111 and ADU-S100 adjuvants induced heterologous and heterosubtypic virus-specific IgG and sIgA titers in both the serum and lungs of pigs.

## 4. Discussion

Vaccination/immunization is a global health success story that mitigates 3.5–5 million deaths every year from infectious diseases such as pertussis, tetanus, diphtheria, influenza, and measles. Furthermore, human, animal, and plant health are interlinked and interdependent on the health of the ecosystem in which they reside, and this approach is referred to as the One Health approach. Therefore, the development of novel and efficacious vaccines against swine influenza is of critical importance to prevent the emergence of potential pandemics and to secure food security [29]. Previous research from our lab demonstrated the efficacy of a plant-derived Nano11 adjuvant-based mucosal vaccine platform for the delivery of whole inactivated SwIAV killed antigen (KAg) in pigs [13,24], and a split SwIAV vaccine composed of a STING agonist in specific pathogen free (SPF) pigs administered by parenteral routes [17]. The present study characterized the split SwIAV H1N2-OH10 antigens adsorbed with Nano11 (Nano11–SwIAV) and co-adsorbed with ADU-S100 adjuvant (NanoS100–SwIAV) administered intranasally in conventional pigs.

Intranasal vaccination with the NanoS100–SwIAV vaccine led to partial clearance of the heterologous H1N1 challenge virus loads in the upper respiratory tract, while clinical responses were not apparently visible in both of the virus-challenged animals. To elucidate the mechanistic insights in detail, we investigated both innate and adaptive immune recall memory responses at the cellular level. The IFNγ-inducible chemokine receptor CXCL10 expressing myeloid cells recruit CXCR3 expressing activated Th1 lymphocytes to the sites of infection/inflammation [30]. CXCL10 controls viral infection and has a role in innate immune response via the recruitment and activation of natural killer cells and exerts a potent chemotactic effect on activated T lymphocytes by binding CXCR3 [31]. Therefore, we studied the frequencies of CXCL10^+^ dendritic cells and monocytes co-expressing the activation marker CD80/86 in pigs. NanoS100–SwIAV vaccine upon the SwIAV-H1N1-OH7 and H1N1 pandemic virus challenge infection stimulated the population of CXCL10^+^CD80/86^+^-activated monocytes in the draining TBLN and in the systemic compartment (Table 1). This data suggests that the NanoS100–SwIAV vaccine can mediate the activation of innate monocyte responses via CXCL10-dependent pathways.

We determined the challenge virus-specific adaptive memory T cell responses in the lung draining TBLN and blood. Activated T-helper/memory cells and NK cells exhibit enhanced surface expression of β1 integrins such as CD49d in pigs [28], and hence, we used CD49d as a surrogate marker for antigen-experienced cells in flow cytometry analysis. Both the Nano11–SwIAV and NanoS100–SwIAV vaccines augmented the frequencies of challenge virus-specific T cell responses in pigs (Table 1).

Total IL-17A^+^ and IFNγ^+^ and antigen responsive CD49d^+^IL-17A^+^ and CD49d^+^IFNγ^+^ CTLs in TBLN MNCs are shown in Table 1. While in the systemic compartment (PBMCs), although both NanoS100-SwIA and Nano11–SwIAV vaccines enhanced the numbers of IFNγ^+^ CTLs, only the NanoS100–SwIAV vaccine upregulated the frequencies of CD49d^+^IFNγ^+^ antigen responsive CTLs in H1N1 pandemic virus-challenged animals (Table 1). These data suggest that NanoS100–SwIAV is stimulating both systemic and mucosal H1N1 pandemic virus-specific IL-17A and IFNγ dominant CTLs responses. The underlying mechanisms that are responsible for these differences need detailed investigation. Hence, it is likely that the immunodominant antigenic epitopes in SwIAV-H1N1-OH7 and 2009 H1N1 pandemic viruses are different, and thus, specific protective T-helper/memory cells and CTLs responses vary substantially. Furthermore, it is possible that the combination adjuvants ADU-S100 and Nano11 stimulate antigen cross-presentation [32] and hence the induction of strong antigen-specific T cell responses following H1N1 pandemic virus challenge infection. These data emphasize the significance of the stimulation of antigen-specific adaptive memory responses leading to long lasting protection. Consistent with our observations, the vaccine virus used in our study SwIAV H1N2-OH10 and the two challenge viruses SwIAV-H1N1-OH7 and 2009 H1N1 pandemic virus are heterologous, with 77% HA gene identity [20,21,22]. Furthermore, the SwIAV-H1N1-OH7 and H1N1 pandemic virus are heterologous to each other, with 93.5% HA gene identity.

Our data underline the protective importance of antigen-specific adaptive immunity in influenza infection, and this is consistent with the published literature. Previous work from the lab established the phenotypes of innate, adaptive, and regulatory immune cells during the acute phase of SwIAV H1N1-OH7 infection in pigs [33]. The significant proliferation of virus-specific CD8^+^ T lymphocytes was documented in TBLN, BAL fluid, lungs, and tonsils at DPC6 [33]. In addition, other research groups have also reported the significant augmentation of antigen-specific CD8^+^ T lymphocyte frequencies in both BAL fluid and blood of SwIAV H1N1-OH7 and H1N1 pandemic virus infection in pigs [34,35]. Furthermore, the role of antigen-specific CD8^+^ T lymphocytes in conferring heterosubtypic immunity has been documented [34]. Consistent with this, Talker and co-workers (2016) characterized the kinetics, phenotype, differentiation status, ability to generate multiple cytokines (multifunctionality), and cross-reactivity of SwIAV-H1N2-specific porcine T lymphocytes, suggesting their potential protective role. This study documented the early effector phenotype of activated and proliferating antigen-specific CD8^+^ T lymphocytes at day 6 post-infection (DPI). Furthermore, starting from DPI4, antigen-specific CD4^+^ and CD8^+^ T lymphocytes generated mainly IFNγ and/or TNF-α and attained maximum peak frequencies at DPI9 in the lungs rather than in the TBLN and blood. These cells also exhibited cross-reactivity to heterologous influenza virus strains [36]. In a study in mice [37], following baculovirus-derived virus-like particle (VLP) intranasal vaccination, observed antigen-specific CD8^+^ T lymphocytes mediated protection against homo- and heterosubtypic IAV infections. Likewise, another study also documented the evidence for the protective significance of cross-protective (heterosubtypic immunity) lung-resident memory CD8^+^ T cells following intramuscular vaccination with a tandem repeat extracellular domain of M2 (M2e) epitopes on VLP [38]. Using a ferret model, Reber and co-workers (2018) investigated the extent of T cell cross-reactivity between contemporary and historical influenza strains upon prime-boost intramuscular (IM) vaccination and infection with H1N1 pandemic virus or the seasonal H3N2 strain. The most potent cross-reactivity was targeted towards peptides from nucleoprotein (NP) in both CD4^+^ and CD8^+^ T cells. Furthermore, hemagglutinin (HA) and neuraminidase (NA) epitopes were also targeted by cross-reactive CD4^+^ T cells. However, CD8^+^ T cells targeted internal matrix protein (M1), nonstructural protein 2 (NS2), and acid polymerase (PA) epitopes [39]. Our experimental results underscore the protective significance of antigen-specific cross-reactive T cell responses against conserved epitopes in influenza infection, suggesting that, in our study, the split virus antigen delivered via Nano11 and STING adjuvant likely presented multiple conserved epitopes on both the H1N1 influenza viral surface and internal viral proteins.

With respect to humoral memory responses, intranasal administration of NanoS100–SwIAV upon SwIAV H1N1-OH7 challenge elicited significantly high cross-protective SwIAV H1N1-OH7- and H3N2-OH4-specific sIgA titers in the lungs. However, in the case of the H1N1 pandemic virus challenge, NanoS100–SwIAV stimulated significant titers of H1N1 pandemic virus- and SwIAV-H1N2-OH10-specific serum and lung IgG and lung sIgA and SwIAV H3N2-OH4-specific lung sIgA, compared to their Mock+Ch counterparts. The experimental animals were raised conventionally, reflecting field conditions, with maternal influenza-specific IgG antibodies of various levels as sows were vaccinated. Therefore, we detected high basal/pre-existing immune responses in the animals. The DPC0 data confirm this observation (Supplementary Figure S2). Hence, it is obvious that the two Nano11-based vaccines elicited cross reactive antigen-specific humoral immune responses.

Our results demonstrate that NanoS100–SwIAV can efficiently induce the expression of CD49d in both T-helper/memory cells and CTLs in both the lung draining TBLN and systemic compartment. Furthermore, CD49d, due to its ability to act as a signaling receptor, has been shown to augment B cell survivability via the upregulation of the expression of Bcl-2 family members [40,41]. Hence, it is possible that NanoS100–SwIAV is boosting antigen-specific B cell responses, resulting in the generation of virus-specific IgG and sIgA titers in conventional pigs. Further investigation is required to investigate the specific memory B cell frequencies in the TBLN and bone marrow of pigs to delineate the vaccine-induced responses.

The above described findings are in agreement with published work on split virus influenza vaccines. In a study in humans, high-dimensional proteomic profiling by mass-cytometry and Luminex was employed to characterize major signaling pathways and cytokine production for all major immune cells in whole-blood stimulated with split virus influenza vaccine (SVIV). SVIV treatment ex vivo stimulated an overlapping but unique proteomic signature profile, different from Toll-like receptor (TLR) activation. The stimulatory activity of SVIV was dependent on the presence of influenza-specific antibodies, suggesting that this stimulatory activity was immune complex-dependent, mediated via Fcγ receptors. Hence, persons with pre-existing immunity mount potent antigen-specific immune responses [19]. Another important aspect is that splitting disrupts the intrinsic adjuvanticity of SVIV, such as RNA. Therefore, combining SVIV with potent adjuvants is essential to ensure a robust induction of cross-protective antigen-specific responses. In the present study, the combination adjuvant comprising Nano11 [13,24] and a synthetic STING agonist ADU-S100 [42,43] is very potent and hence, upon combination with split virus SwIAV-H1N2-OH10 antigens, elicits strong protective antigen-specific immune responses. Zhang and co-workers (2021) reported that intradermal administration of heat shock protein gp96-adjuvanted seasonal influenza H1N1 monovalent split vaccine in mice leads to the induction of antigen-specific broadly cross-protective CD8^+^ T cells [44]. In contrast, intranasal administration of the split trivalent influenza vaccine to mice with anionic adjuvant endocrine or cationic adjuvant N3OA elicited significantly augmented antibody and cell-mediated responses, compared with the non-adjuvanted vaccine, while the other cationic adjuvant N3OAsq significantly increased cell-mediated immune responses only. Furthermore, intranasal vaccination of the influenza vaccine in combination with any of the adjuvants, either endocrine N3OA or N3OASq, elicited the mucosal immunity against influenza HA protein [45].

Various research groups have documented the therapeutic role/s of antigen-specific Th17 responses. Subcutaneous administration of a trivalent subunit influenza vaccine with a cationic liposome adjuvant system CAF01 augmented the antigen-specific humoral immunity, robust Th1, and Th17 responses in mice. Furthermore, Th1/Th17 cytokines were persistent until 20 weeks after the last vaccination. In addition, CAF01-adjuvanted influenza vaccine conferred complete protection of mice challenged with a drifted H1N1 influenza strain [46]. In another study in mice, pulmonary vaccination with modified *Mycobacterium bovis* bacillus Calmette–Guerin (BCG) vaccine resulted in suppression of inflammation, prevented lung immunopathology, and conferred significant protection against *M. tuberculosis* infection, and this protection was directly linked to IL-17A response [47]. In an experimental human pneumococcal carriage model, the researchers documented the protective role of Th17 memory responses in the lungs, and in vitro augmentation of rhIL-17A stimulated IL-17RA^+^ alveolar macrophage-mediated killing of opsonized pneumococci [48]. In addition, in a swine model of enterotoxigenic *Escherichia coli* (ETEC) infection, oral immunization with F4 fimbriae stimulated a robust antigen-specific protective systemic Th17 response [49]. Continuing in the same vein, intradermal administration of NanoS100–SwIAV in SPF pigs elicited significant systemic antigen-specific Th17 and IL-17A^+^ CTL responses compared to their non-adjuvant vaccinate counterparts. Furthermore, the dose sparing effect was observed in the case of intradermal vaccination compared to intramuscular vaccination in SPF pigs [17]. These observations highlight the protective role/s of antigen-specific IL-17A responses. To summarize, although NanoS100–SwIAV elicited strong virus-specific humoral responses, dominant and robust systemic antigen-specific Th1 and Th17 responses were upregulated (Table 1). However, in the lung draining TBLN, both NanoS100–SwIAV and Nano11–SwIAV vaccine formulations were efficacious in stimulating antigen-specific Th1 and Th17 responses. These data emphasize the critical role played by cross-protective antigen-specific cell-mediated memory responses against SwIAV infection.

Although the current pool of serotype-specific influenza vaccines mainly elicits antibody responses against viral surface proteins such as HA and NA, they are deficient in the induction of cell-mediated responses targeting viral conserved internal proteins. Furthermore, antigenic shift and drift contribute toward the constant evolution of field strains and the evasion of host immune responses. Hence, the stimulation of humoral responses against receptor-binding epitopes and broadly neutralizing conserved epitopes, coupled with robust cell-mediated immune responses against highly conserved internal proteins, is a better strategy. The conserved proteins NP, Matrix protein 2 (M2), and relatively conserved HA stem region have been reported to confer broad cross-protection/heterosubtypic immunity in preclinical animal models [50]. Another important aspect is the current criteria for influenza vaccine licensing which mainly consider serum antibody titers determined by hemagglutination inhibition (HI) assay, single radial hemolysis (SRH), and a virus microneutralization test, identifying different classes of antibodies, and therefore, there is a variation in the extent of correlation. These tests have several limitations such as low sensitivity, high variability between labs, lack of suitability for live attenuated influenza virus evaluation, and lack of standardized protocols. In addition, current correlates of protection consider healthy individuals but not susceptible subjects such as young children and older adults. Therefore, there is a pressing need for the establishment of novel correlates of protection involving cell-mediated immune responses. In addition, novel vaccines such as DNA/mRNA vaccines need novel correlates of protection to assay the vaccine efficacy. Via simultaneous measurement of the surface, intracellular markers, and cytokines/chemokines using flow cytometry or enzyme linked immunoSPOT (ELISPOT) assays, we can determine changes in phenotype, activation statuses, and differentiation stages of T and B lymphocytes, leading to the determination of novel correlates of protection [51]. Furthermore, the host innate immune system exhibits a unique type of memory, known as trained immunity, which is characterized by a relatively long-term adaptation mediated by steady epigenetic alterations and metabolic reprogramming of innate immune cells [52,53]. Research efforts should tap the immense potential of trained immunity leading to the rational vaccine design strategies against evolving pathogens.

Upcoming research activities are aimed at elucidating the mechanisms of mucosal vaccine efficacy involving multivalent SwIAV vaccine candidates in combination with potent adjuvants. Furthermore, delineation of the interplay of the host and microbiota, mucosal routes of vaccination, and adjuvants by using unbiased systems of biology strategies are essential to combat the threat of constantly evolving SwIAV field strains and potential pandemics.

## 5. Conclusions

Irrespective of the vast genetic difference (77% HA gene identity) between the vaccine H1N2 and both of the H1N1 challenge influenza viruses, intranasal vaccination of split virus antigens surface adsorbed onto Nano11 particles, along with the STING adjuvant combination, elicited superior cross-protective T cell responses. This was observed both in the mucosal and systemic immune compartments, suggesting the utility of the combination of mucosal adjuvants in mitigating SwIAV infection in pigs and in reducing the transmission of zoonotic SwIAV from pigs to humans.

## Figures and Tables

**Table 1:** Summary of significantly modulated myeloid and lymphoid immune cells in Nano11-based vaccinated pigs.

Phenotypes of Ivmohoid and Maeloid Cells T-Helper/Memory Cells [CD3^+^CD4^+^CD8α^+^CD8β^−^] CTLs [CD3^+^CD4^−^CD8α^+^CD8β^+^] Monocytes [CD3^−^CD17a^+^SynCAM^−^]	Vaccine Type	Challenge: SwlAVH1N1-OH7	Challenge: H1N1 Pandemic
TBLN MNCs: CXCL10^+^CD80/86^+^ activated monocytes	NanoS100-SwlAV	*** *p* < 0.001	** *p* < 0.01
	Nano11-SwlAV	*** *p* < 0.001	NS
TBLN MNCs: CD49d^+^ antigen responsive T-helper/memory cells	NanoS100-SwlAV	-	-
	Nano11-SwlAV	-	* *p* < 0.05
TBLN MNCs: IFNγ^+^ antigen activated CTLs	NanoS100-SwlAV	*** *p* < 0.05	-
	Nano11-SwlAV	** *p* < 0.01	-
TBLN MNCs: CD49d^+^IFNγ^+^ antigen responsive CTLs	NanoS100-SwlAV	** *p* < 0.01	-
	Nano11-SwlAV	* *p* < 0.05	-
TBLN MNCs: IL-17A^+^ antigen activated CTLs	NanoS100-SwlAV	NS	** *p* < 0.01
	Nano11-SwlAV	* *p* < 0.05	* *p* < 0.05
TBLN MNCs: CD49d^+^IL-17A^+^ antigen responsive CTLs	NanoS100-SwlAV	-	** *p* < 0.01
	Nano11-SwlAV	* *p* < 0.05	** *p* < 0.01
PBMCs: IFNγ^+^ antigen activated CTLs	NanoS 100-SwlAV	-	** *p* < 0.01
	Nano11-SwlAV	-	* *p* < 0.05
PBMCs: CD49d^+^IFNY^+^ antigen responsive CTLs	NanoS100-SwlAV	-	** *p* < 0.01
	Nano11-SwlAV	-	-

Significantly enhanced immune cell subsets in both NanoS100–SwIAV and Nano11–SwIAV vaccinates following SwIAV-H1N1-OH7 or H1N1 pandemic virus challenge infection compared to their control groups at DPC6. Asterisks represent significant difference between indicated groups (* *p* < 0.05, ** *p* < 0.01, *** *p* < 0.001).

## Data Availability

No new data were created or analyzed in this study. Data sharing is not applicable to this article.

## Supplementary Materials

The following supporting information can be downloaded at: https://www.mdpi.com/article/10.3390/vaccines11111707/s1, Figure S1: Representative gating strategies for the analysis of T-lymphocytes and myeloid cells in PBMCs. Conventional pigs were immunized twice with Nano11-SwIAV or NanoS100-SwIAV split virus vaccine or controls Mock IN and challenged at day post-prime vaccination 35 with SwIAV H1N1-OH7 or H1N1 pandemic virus and euthanized at day post challenge 6 (DPC6). PBMCs, TBLN MNCs, and BAL cells isolated at DPC6 were restimulated with SwIAV H1N1-OH7 or H1N1 pandemic virus in vitro. The cells were immunolabeled and analyzed by flow cytometry for the frequencies of different types of myeloid and lymphocyte subsets. (A) CD80/86^+^CXCL10^+^ and total CD80/86^+^ dendritic cells/monocytes; (B) CD49d^+^IL-17A^+^ CTLs and T-helper/memory cells; (C) Total and CD49d^+^IFNγ^+^ CTLs. Figure S2: SwIAV-specific antibody responses at post vaccination day 35 (DPC0). Conventional pigs were immunized twice with Nano11-SwIAV or NanoS100-SwIAV split virus vaccine or controls Mock. Serum IgG titers were determined against (A) H1N1 pandemic; (B) H1N1-OH7; (C) H1N2-OH10; (D) H3N2-OH4; and Nasal swab (NS) sIgA titers were measured against (E) H1N1 pandemic; (F) H1N1-OH7; (G) H1N2-OH10; (H) H3N2-OH4. Data represent the mean value of 5 or 6 pigs ± SEM. Statistical analysis was performed by two-way ANOVA followed by Bonferroni post-test. Letters a, b, c refers to significance between Mock versus Nano11-SwIAV, Mock versus NanoS100-SwIAV, and Nano11-SwIAV versus NanoS100-SwIAV, respectively. Tables S1–S3: Antibody panels used in flow cytometry analysis. The antibody panels employed for the analysis of T-helper/memory cells, CTLs, and myeloid cells in TBLN MNCs, PBMCs, and BAL cells in Panel#1 [Table S1] (Myeloid cells); IL-17A+ lymphocyte subsets in Panel#2 [Table S2]; and IFNγ+ lymphocyte subsets in Panel#3 [Table S3].
