# Supplementary material for: Characterization of the Efficacy of a Split Swine Influenza A Virus Nasal Vaccine Formulated with a Nanoparticle/STING Agonist Combination Adjuvant in Conventional Pigs
Source: Vaccines (Basel). 2023 Nov 10;11(11):1707. doi: 10.3390/vaccines11111707 (PMC10675483; doi:10.3390/vaccines11111707)
Supplement: Supplementary file 1 [file vaccines-11-01707-s001.zip › vaccines-2627306-supplementary.pdf]

Supplementary Table S1

	Panel#1	Isotype	Concentration used
1	Mouse anti-porcine CD3 AF488 Clone PPT3 (SouthernBiotech)	Mouse IgG1κ	1 µg/ml
2	Mouse anti-porcine CD172a-Biotin Clone 74-22-15 (SouthernBiotech)	Mouse IgG1κ	0.5 µg/ml Streptavidin-PE Texas Red (1:750 dilution)
3	Human CTLA-4 Ig PE (Ancell)	Mouse IgG2a	1:100 dilution
4	Mouse anti-porcine CD4 unlabeled Clone 74-12-4 (SouthernBiotech) Labeled with AF700 (Biotium)	Mouse IgG2b κ	6 µg/ml
5	Chicken anti-mouse SynCAM unlabeled Clone 3E1 (MBL) Labeled with APC/Cy7 (Biotium)	Chicken IgY	0.5 µg/ml
6	Mouse anti-porcine CXCL10 unlabeled Clone 1.4 (Toolkit - Lunney) Labeled with AF647 (Invitrogen)	Mouse IgG1κ	7 µg/ml

Supplementary Table S2

	Panel#2	Isotype	Concentration used
1	Mouse anti-porcine CD3 AF488 Clone PPT3 (SouthernBiotech)	Mouse IgG1κ	1 µg/ml
2	Mouse anti-porcine CD8α-Biotin Clone 76-2-11 (SouthernBiotech)	Mouse IgG2aκ	0.5 µg/ml Streptavidin-PE Texas Red (1:750 dilution)
3	Mouse anti-porcine CD8β unlabeled Clone PG164A (Monoclonal Antibody Center-Washington State University)	Mouse IgG2a	0.5 µg/ml Goat anti-mouse IgG2a APC/Cy7 secondary antibody (SouthernBiotech)
4	Mouse anti-porcine CD4 unlabeled Clone 74-12-4 (SouthernBiotech) Labeled with AF700 (Biotium)	Mouse IgG2b κ	6 µg/ml
5	Mouse anti-porcine IL-17A unlabeled Clone 1.1 Labeled with AF647 (Invitrogen)	Mouse IgG1κ	7 µg/ml
6	Mouse anti-human CD49d PE Clone L25	Mouse IgG2b	2.0 µl/test

Supplementary Table S3

	Panel#3	Isotype	Concentration used
1	Mouse anti-porcine CD3 AF488 Clone PPT3 (SouthernBiotech)	Mouse IgG1κ	1 µg/ml
2	Mouse anti-porcine CD8α-Biotin Clone 76-2-11 (SouthernBiotech)	Mouse IgG2aκ	0.5 µg/ml Streptavidin-PE Texas Red (1:750 dilution)
3	Mouse anti-porcine CD8β unlabeled Clone PG164A (Monoclonal Antibody Center-Washington State University)	Mouse IgG2a	0.5 µg/ml Goat anti-mouse IgG2a APC/Cy7 secondary antibody (SouthernBiotech)
4	Mouse anti-porcine CD4 unlabeled Clone 74-12-4 (SouthernBiotech) Labeled with AF700 (Biotium)	Mouse IgG2b κ	6 µg/ml
5	Mouse anti-porcine IFNγ AF647 Clone P2G10 (BD Bioscience)	Mouse IgG1κ	1 µg/ml
6	Mouse anti-human CD49d PE Clone L25	Mouse IgG2b	2.0 µl/test

Supplementary Tables S1-S3. Antibody panels used in flow cytometry analysis. The antibody panels employed for the analysis of T-helper/memory cells, CTLs, and myeloid cells in TBLN MNCs, PBMCs, and BAL cells in Panel#1 [Table S1] (Myeloid cells); IL-17A^{+} lymphocyte subsets in Panel#2 [Table S2]; and IFNγ^{+} lymphocyte subsets in Panel#3 [Table S3].

Supplementary Fig. S1A Gating strategy for the analysis of CD3$^-$CD172a$^+$SynCAM$^-$ monocytes and CD3$^-$CD172a$^+$SynCAM$^+$ dendritic cells in PBMCs (Panel#1)

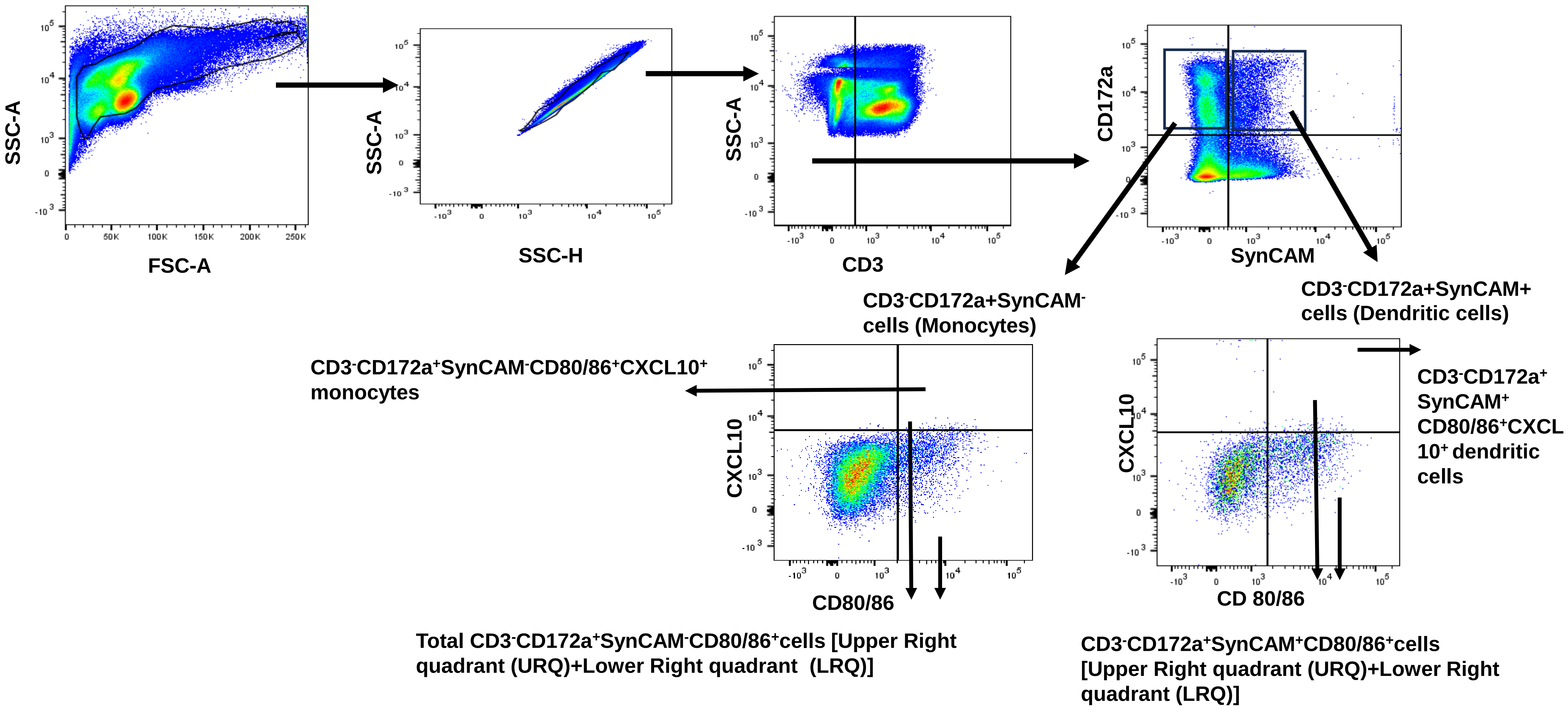

Supplementary Fig. S1B Gating strategy for the analysis of CD49d±/ IL-17A± T-lymphocytes in PBMCs at DPC6 (Panel#2)

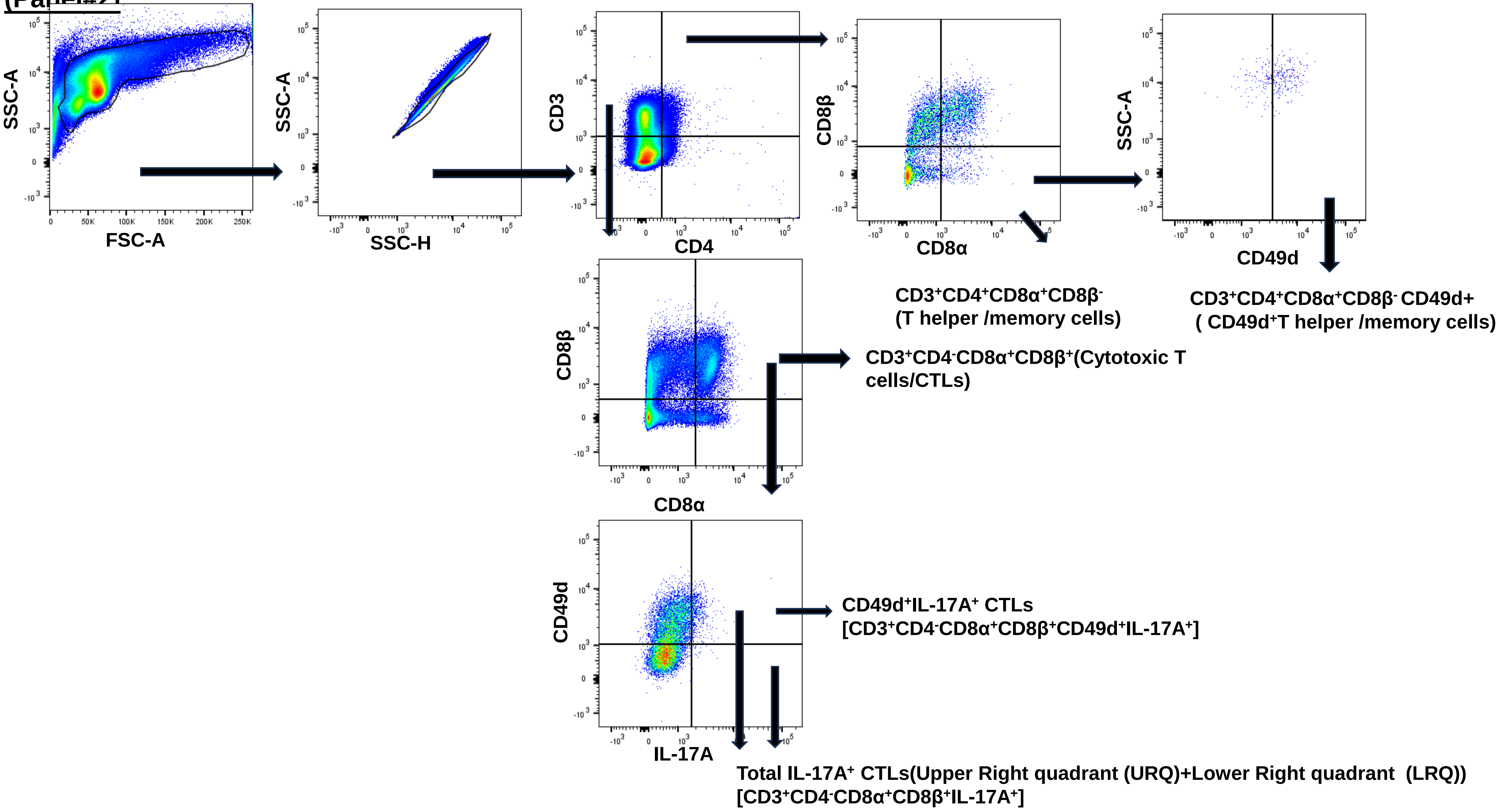

Supplementary Fig. S1C Gating strategy for the analysis of CD49d±/IFNγ± CTLs in PBMCs at DPC6 (Panel#3)

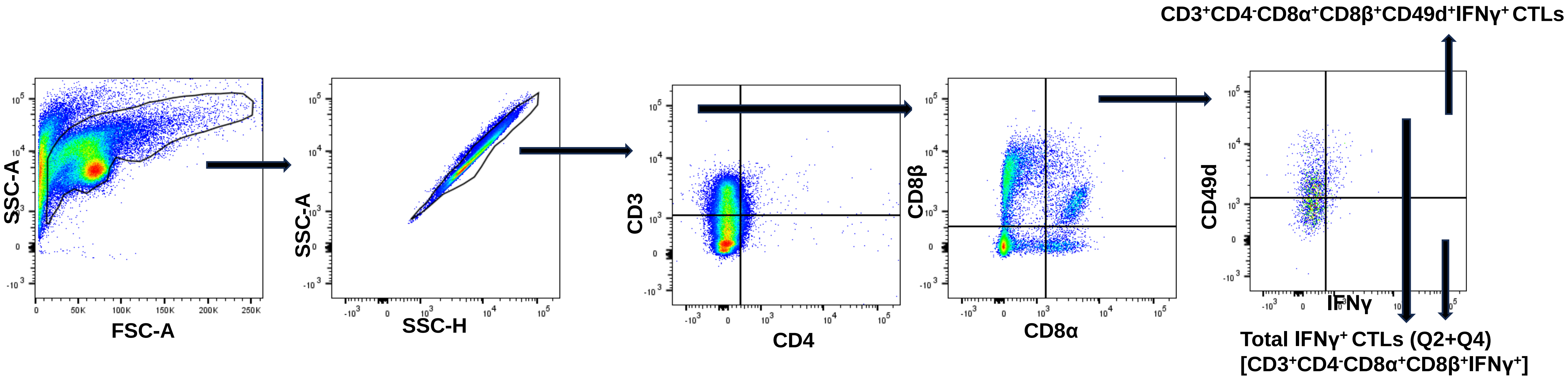

Supplementary Figure S1. **Representative gating strategies for the analysis of T-lymphocytes and myeloid cells in PBMCs.** Conventional pigs were immunized twice with Nano11-SwIAV or NanoS100-SwIAV split virus vaccine or controls Mock IN and challenged at day post-prime vaccination 35 with SwIAV H1N1-OH7 or H1N1 pandemic virus and euthanized at day post challenge 6 (DPC6). PBMCs, TBLN MNCs, and BAL cells isolated at DPC6 were restimulated with SwIAV H1N1-OH7 or H1N1 pandemic virus *in vitro*. The cells were immunolabeled and analyzed by flow cytometry for the frequencies of different types of myeloid and lymphocyte subsets. **(A)** CD80/86^{+}CXCL10^{+} and total CD80/86^{+} dendritic cells/monocytes; **(B)** CD49d^{+}IL-17A^{+} CTLs and T-helper/memory cells; **(C)** Total and CD49d^{+} IFNγ^{+} CTLs.

Supplementary Fig. S2 SwIAV-specific antibody responses at post vaccination day 35 (DPC0)

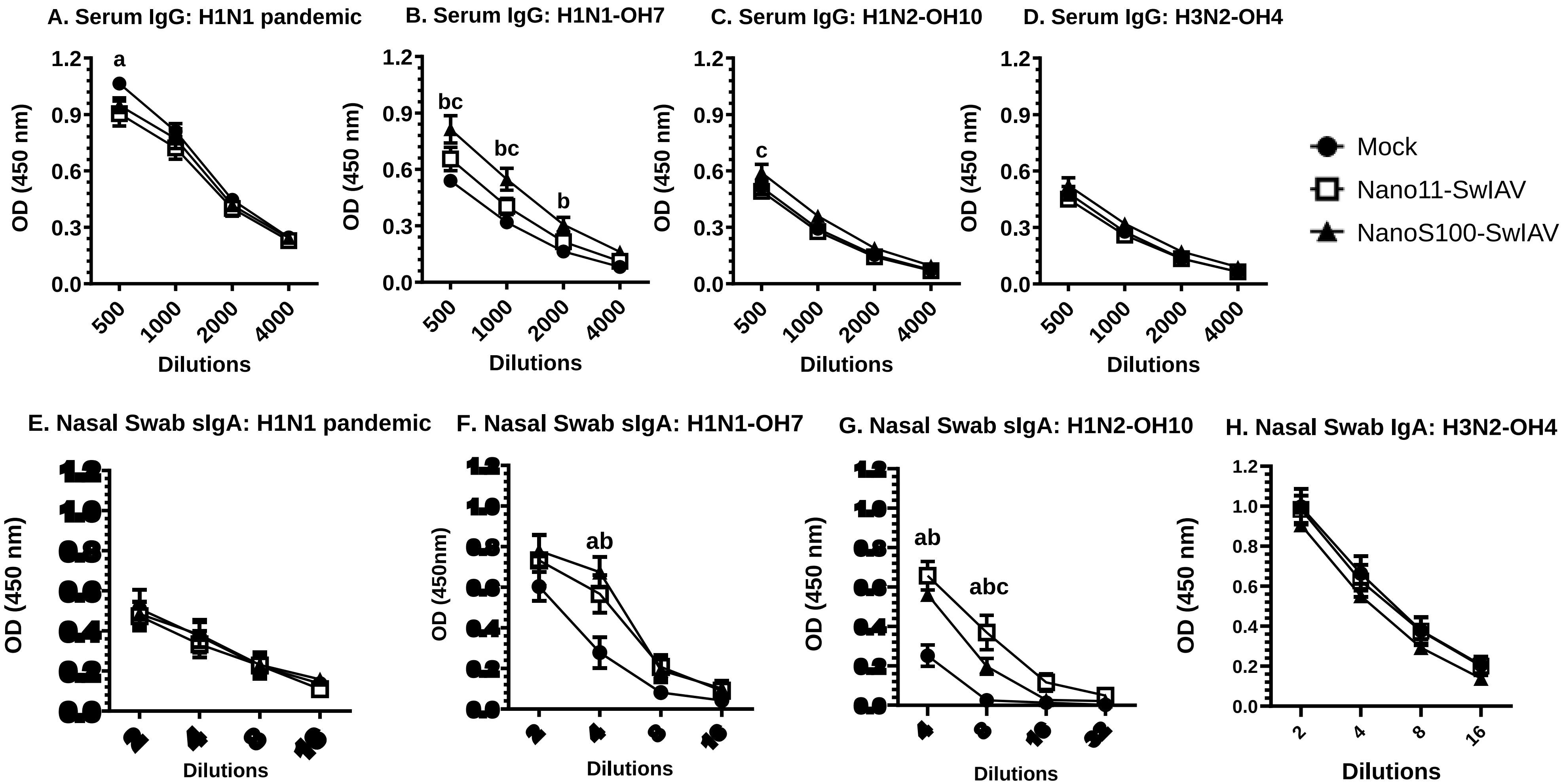

Supplementary Figure S2. SwIAV-specific antibody responses at post vaccination day 35 (DPC0). Conventional pigs were immunized twice with Nano11-SwIAV or NanoS100-SwIAV split virus vaccine or controls Mock. Serum IgG titers were determined against (A) H1N1 pandemic; (B) H1N1-OH7; (C) H1N2-OH10; (D) H3N2-OH4; and Nasal swab (NS) sIgA titers were measured against (E) H1N1 pandemic; (F) H1N1-OH7; (G) H1N2-OH10; (H) H3N2-OH4. Data represent the mean value of 5 or 6 pigs ± SEM. Statistical analysis was performed by two-way ANOVA followed by Bonferroni post-test. Letters a, b, c refers to significance between Mock versus Nano11-SwIAV, Mock versus NanoS100-SwIAV, and Nano11-SwIAV versus NanoS100-SwIAV, respectively.
